# Investigations on the complex flows induced by dual-swept/dual-ramp wedges in supersonic flows

**DOI:** 10.1038/s41598-020-66676-5

**Published:** 2020-06-12

**Authors:** X. Gao, G. X. Xiang, W. J. Tang, X. Z. Jie, X. Huang, J. Y. He, S. A. Liu

**Affiliations:** 1 College of Geology and Environment, Xi’an University of Science and Technology, Xi’an, 710000 China; 2 School of Mechanics, Civil Engineering & Architecture, Northwestern Polytechnical University, Xi’an, 710129 China; 3 China Coal Research Institute, China Coal Technology & Engineering Group, Beijing, 100013 China; 4 Beijing Institute of Land Renovation and Ecological Restoration, China Coal Technology & Engineering Group, Beijing, 100013 China

**Keywords:** Fluid dynamics, Aerospace engineering

## Abstract

The caret inlet with a dual-swept/dual-ramp configuration has excellent stealth performance and aerodynamic capability. Most previous investigations on this configuration have focused on experiments and numerical simulations but there are relatively few theoretical investigations. In this study, the flow field characteristics of dual-swept/dual-ramp configuration are investigated analytically and numerically. An analytical approach that combines the shock dynamics with a “spatial dimension reduction” was used to analyze the characteristics of the wave structures and state parameters of the flow field. The effects of the sweep angles and inflow Mach number on the flow field characteristics are investigated. The results indicate that the problem of shock/shock interaction in two intersecting wedges of large back-swept angle is a problem of weak shock interaction. Therefore, the theory of weak shock interaction is used to investigate the flow field characteristics, including the uniformity of the flow field and the total pressure recovery performance.

## Introduction

In aerospace engineering, the effective design of modern supersonic and hypersonic vehicles requires thorough understanding of the physical flowfield structure of shock/shock interaction^1–10^. The model of two intersecting wedges in supersonic or hypersonic flow has been widely used in aerospace engineering^11–25^. A typical application of two intersecting wedges in supersonic flow is the caret inlet, which provides compressed air by two back-swept intersecting wedges. The caret inlet meets the requirements of modern fighters, including a simple structure, good stealth performance, and good maneuverability. The caret inlet was first used in the U.S. carrier fighter F/A-18E/F^11^ and the test results proved that the total pressure recovery performance was better for the caret inlet than the conventional two-dimensional inlet^12^. Therefore, an understanding of the flow field of the two back-swept corners is of great importance for the designs of such inlets.

Teng and Settles^13,14^ investigated the two back-swept intersecting wedges and conducted a series of experiments on caret inlets with back-swept compression surfaces; the results indicated the existence of a three-dimensional interactive zone in the vicinity of the apex. Subsequently, Horstman and Settles^15,16^ investigated the wave structures of the flow field induced by two intersecting sharp wedges; the results indicated that the wave configurations were conical and self-similar. Further investigations by Dolling and Settles^17,18^ indicated that the inviscid parameters dominated the interacting flow field and that the effects of the viscosity were negligible. Zhu^19,20^ conducted experiments and numerical simulations to investigate the aerodynamic characteristics of a caret inlet and found that the total pressure recovery rapidly decreased from Mach 1.6 to 2.0. He^21^ studied the wave configuration at the entrance of a caret inlet numerically and investigated the effects of the sweep angle, compression angle, and inflow Mach number on the wave configurations. Zhong^22,23^ conducted experiments on the gas-dynamic performance of a caret inlet under the conditions of a low inflow Mach number, a large attack angle, and a large yaw angle. However, the above-mentioned studies mostly focused on experiments and numerical simulations and relatively few theoretical studies have been conducted on caret inlets.

In this study, an analytical approach called “spatial dimension reduction” is used to solve the problem of the three-dimensional (3D) shock/shock interactions (SSI) with dual-swept intersecting wedges; this is a significant problem in caret inlets^24,25^. Numerical simulations are conducted to validate the theoretical results and investigate the flow field characteristics in caret inlets. The paper is organized as follows. In the second section, we describe the concept of the “spatial dimension reduction” in caret inlets and the numerical method. In the third section, we elaborate on the combination of the theoretical and numerical methods to investigate the flow field characteristics in two intersecting swept wedges and caret inlets; both the wave configurations and state parameters are solved analytically and numerically. The effects of the back-sweep angle on the wave configurations and flow field characteristics are described in the part *A* of the third section. In the part *B* of the third section, the mechanisms of the SSI in the caret inlet are explained and several caret inlets are designed and investigated. Finally, the conclusions are drawn in Section 4.

## Theoretical approach and numerical methods

**Figure 1:**
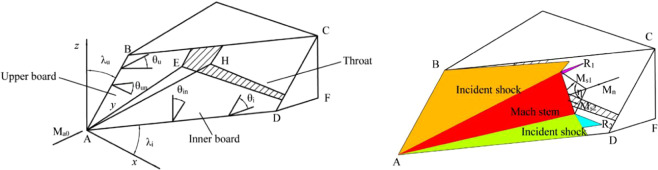
Schematic of Caret inlet.

Figure 1 shows the model and the wave structure of a Caret inlet with dual-swept/dual-ramp configuration. The flows are compressed by the inner board *ADFE* and the upper board *ABE* and the entrance is a spatial parallelogram *ABCD*. The direction of the incoming flow is parallel to *AE* and *CFH* is the inlet throat. The back-sweep angle and the wedge angle of the inner board are *λ*_*i*_ and *θ*_*i*_, whereas the back-sweep angle and the wedge angle of the upper board are *λ*_*u*_ and *θ*_*u*_. The effective compression angles *θ*_*in*_ and *θ*_*un*_ are the wedge angles that are perpendicular to leading lines *AD* and *AB*. For the two boards, two incident shocks are formed attached to the wedges and a Mach stem is formed between two incident shocks.

The relationships between the effective compression angles and wedge angles are as follows:

1 $$\tan \,{\theta }_{u}=\,\tan \,{\theta }_{un}\,\cos \,{\lambda }_{u},\,\tan \,{\theta }_{i}=\,\tan \,{\theta }_{in}\,\cos \,{\lambda }_{i}$$

For the incoming flow *M*_*a0*_ that is parallel to *AE*, two incident shocks *M*_*s*1_ and *M*_*s*2_ are formed beyond the inner and upper board. *β*_*i*_ and *β*_*u*_ are the shock angle and *β*_*in*_ and *β*_*un*_ are the effective shock angle that is perpendicular to the leading lines. The relationships between the effective shock angles and shock angles are as follows:

2 $$\tan \,{\beta }_{u}=\,\tan \,{\beta }_{un}\,\cos \,{\lambda }_{u},\,\tan \,{\beta }_{i}=\,\tan \,{\beta }_{in}\,\cos \,{\lambda }_{i}$$

**Figure 2:**
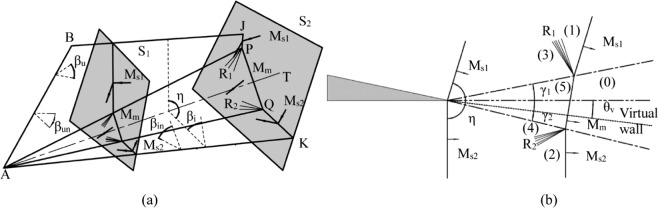
Schematic of “spatial-dimension reduction” approach.

*M*_*s*1_ and *M*_*s*2_ interact with each other and form a Mach stem *M*_*m*_ between them, including two reflected compression waves *R*_1_ and *R*_2_. The intersecting line of the two incident shocks *M*_*s1*_ and *M*_*s2*_ is defined as the characteristic line and the plane perpendicular to the characteristic line is defined as the characteristic plane. As is depicted in Fig. 2(a), the wave structure of the characteristic plane is self-similar. As the cross-sections move in the positive direction of the *y*-axis, the Mach stem increases in length and the distance between the two incident shock planes widens, which is very similar to the two incident waves *M*_*s1*_ and *M*_*s2*_ moving and interacting with each other in a two-dimensional (2D) unsteady flow (Fig. 2(b)). Therefore, the 3D wave configuration at the entrance of the caret inlet can be treated as a 2D wave structure in the characteristic plane moving in the direction of the characteristic line. The dimension of the characteristic line direction is replaced by the time dimension. The detailed procedure of the “spatial dimension reduction” approach has been previously published^26–28^.

After transforming the 3D steady problem to a 2D unsteady problem, the 2D flow field can be solved by the shock dynamics and shock theory^29,30^.

3 $$\tan \,{\theta }_{v}=\frac{{M}_{bs}}{{M}_{sws}}\times \frac{{[1-{({M}_{sws}/{M}_{bs})}^{2}]}^{1/2}\{1-{[f({M}_{bs})/f({M}_{sws})]}^{\frac{1}{2}}\}}{1+f({M}_{bs}){M}_{bs}/f({M}_{sws}){M}_{sws}}$$

4 $$\tan (\pi -\eta -{\theta }_{v})=\frac{{M}_{bs}}{{M}_{rws}}\times \frac{{[1-{({M}_{rws}/{M}_{bs})}^{2}]}^{1/2}\{1-{[f({M}_{bs})/f({M}_{rws})]}^{\frac{1}{2}}\}}{1+f({M}_{bs}){M}_{bs}/f({M}_{rws}){M}_{rws}}$$

The wave configurations can be determined by a shock polar analysis of the 2D unsteady problem. The state parameters such as the pressure, total pressure recovery coefficient, temperature, and density are the same as for the 2D unsteady solutions. The vector parameters, such as the velocities and Mach number, should be combined with the decomposed vectors in the direction of the characteristic line.

In this study, we use a dispersion-controlled dissipation scheme^31^ to solve the 3D inviscid Euler equations; this is a type of total variation diminishing (TVD) scheme that is commonly used^32–52^. The computational mesh is an orthogonal uniformly structured mesh with a mesh quantity of about 8 million. Prior to conducting the numerical simulations, mesh independence tests were performed to ensure that the results are independent of the type of mesh chosen for the numerical simulations. The inlet boundary of the computational zone has a fixed supersonic inflow condition, the far-field boundaries have non-reflecting boundary conditions, and the wall boundaries have solid slipping conditions.

## Results and discussion

As the 3D effects of the two intersecting wedges mostly affect the sweep angle of the wedges, it is difficult to solve the problem of two swept intersecting wedges as a 2D problem. In the present study, the effects of the back-sweep angle are determined and the flow field characteristics are investigated theoretically and numerically. Specifically, one of the significant applications of dual-swept wedges is the caret inlet, which has excellent flow field uniformity and total pressure recovery performance. The internal mechanisms and design methods of the caret inlet are discussed in detail to provide an effective approach for the design and researches of such inlets.

### Effects of the back-sweep angle

**Figure 3:**
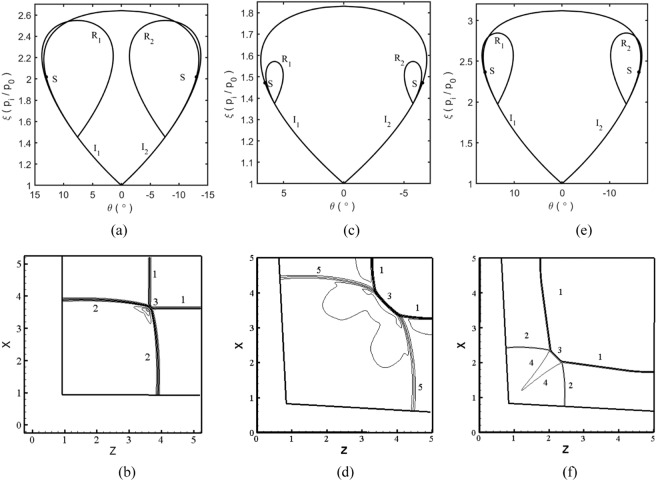
Shock polar analysis and numerical results of two intersecting wedges at (**a,b**) θ_1_ = θ_2_ = 5°, λ_1_ = λ_2_ = 0°, M_0_ = 3, (**c,d**) θ_1_ = θ_2_ = 5°, λ_1_ = λ_2_ = 30°, M_0_ = 3, (**e,f**) θ_1_ = θ_2_ = 5°, λ_1_ = λ_2_ = 30°, M_0_ = 6. Note: *0*-wall, *1*-incident shock, *2*-reflected shock, *3*-internal bridge-shaped shock, *4*-slip line, *5*-compressional waves.

The model of two intersecting wedges with back sweep angles is very common in wing-body combinations and inlets of supersonic or hypersonic vehicles. In order to determine the effects of the back-sweep angle, we use two examples, one with and one without a back-sweep angle. Figure 3a–d presents the analytical and numerical results for the parameters of *θ*_*1*_ = *θ*_*2*_ = 5°, *M*_*a*0_ = 3, and *λ*_1_ = *λ*_2_ = 30° for the back-sweep angles. *P*_*0*_ represents the pressure in the inflow condition, *P*_*i*_ represents the pressure passing through the shock wave. The shock polar analysis shows that the two reflected polars *R*_1_ and *R*_2_ interact with the two incident polars *I*_1_ and *I*_2_ respectively and they do not intersect with each other at *λ*_1_ = *λ*_2_ = 0°. This indicates that a Mach interaction occurs and this is validated by the numerical results. In Fig. 3b, two incident waves *1* interact with each other and two reflected waves *2* and a Mach stem 3 are formed. As the back-sweep angle is increased to 30° (Fig. 3c,d), the two reflected polars *R*_1_ and *R*_2_ are completely inside the two incident polars *I*_1_ and *I*_2_, which indicates that a weak interaction is created. For the weak interaction, the Mach stem 3 is curved and is longer than when there is no back-sweep angle. The reflected wave and the compression wave are observed between the incident wave 1 and the Mach stem 3, which is due to the small disparity of the pressure behind the incident wave and the Mach stem. For the swept wedges, as the inflow Mach number is increased to 6 (Fig. 3e,f), the two reflected polars interact with the two incident polars, indicating that the Mach interaction will occur. Figure 3f shows a typical single Mach interaction, where two incident waves, two reflected waves, one Mach stem, and two slip lines can be clearly seen.

**Figure 4:**
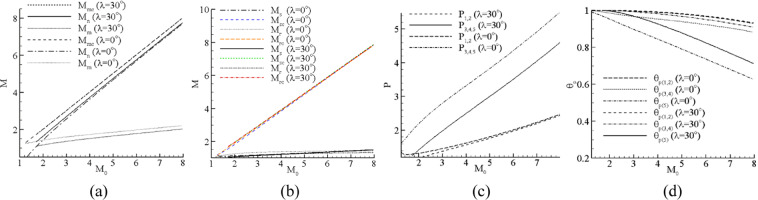
Theoretical results of zones in two intersecting wedges of large sweep angle at θ = 5°, λ = 0°,30°, M0 = 1~8. (**a**) Mach numbers behind the Mach stem, (**b**) Mach numbers behind incident wave and reflected wave, (**c**) static pressure, (**d**) total pressure recovery coefficient.

Figure 4 presents the theoretical results of the flow field for the two intersecting wedges at *θ* = 5°, *λ* = 0°, and *λ* = 30°; the Mach number of the incoming flow *M*_0_ ranges from 1 to 8. Figure 4a,b show the theoretical analysis results of the decomposed Mach numbers of the characteristic plane and the composed Mach numbers of the 3D flow field. *M*_*s*_, *M*_*r*_, and *M*_*m*_ are the Mach numbers of the characteristic plane behind the incident wave, the reflected wave, and the Mach stem respectively. After combining the Mach numbers of the characteristic plane with the Mach number *M*_*n*_ of the characteristic line, the combined Mach numbers *M*_*sc*_, *M*_*rc*_, and *M*_*mc*_ of the 3D flow field are obtained analytically. Figure 4a indicates that the increase in the back-sweep angle results in a decrease in the Mach numbers behind the Mach stem. It should be noted that the Mach number behind the Mach stem *M*_*m*_ at *λ* = 30° are lower than that at *λ* = 0° and the Mach numbers *M*_*n*_ on the characteristic line at *λ* = 30° is higher than that at *λ* = 0°. The combined Mach number *M*_*mc*_ behind the Mach stem in the 3D flow field are almost identical after combining *M*_*m*_ with *M*_*n*_, which means that the changes in the back-sweep angle have little influence on the Mach number behind the Mach stem in the 3D flow. Figure 4b shows that the change in the sweep angle has little influence on the Mach numbers behind the incident wave and reflected wave and the combined Mach numbers behind the incident wave and the reflected wave are almost the same.

Figure 4c,d present the static pressure and the total pressure recovery coefficient in each zone with different inflow Mach numbers and back sweep angles. As the inflow Mach number *M*_*a0*_ increases, the pressure in zones 1–5 increases monotonously and the total pressure recovery coefficient decreases gradually. Because zones 3, 4, and 5 are divided by the slip lines, they have the same pressure (Fig. 4c). As is indicated in Fig. 4d, the total pressure loss is considerably larger behind the Mach stem (dash-dotted line in Fig. 4d) than behind the reflected shock waves (solid line and dotted line in Fig. 4d) because the entropy production that passes through the incident shock waves and reflected shock waves is smaller than that passing through the Mach stem. Figure 4d also shows that the increase in the back-sweep angle decreases the total pressure loss, which is a significant factor in the design of inlets. Even though the combined Mach numbers in the 3D flows are the same for the different back sweep angles, the decomposed Mach numbers, which are perpendicular to the surface of the shock waves, are not the same. Therefore, the changes in the sweep angle affect the flow field parameters, such as the density, temperature, pressure, and the total pressure loss, which is very important to the performance of aircraft and inlets. The static pressure ratio and total pressure recovery coefficient passing through two incident shocks can be obtained by the shock-wave relations.

### Caret inlet

**Figure 5:**
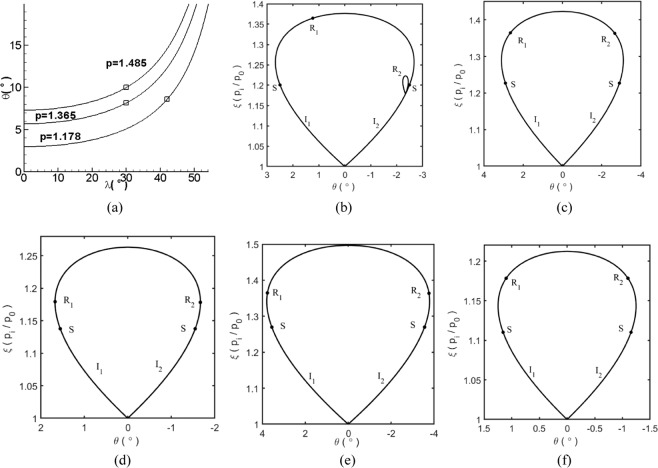
(**a**) Theoretical pressure contour lines with the combinations of different sweep angle and wedge angle at *M*_0_ = 2, (**d–f**) Shock polar analysis for wave configurations of Caret inlet in Table 1.

In order to obtain uniform flow, the combination of sweep angle and wedge angle that produces equal pressure is selected (see the square symbols on each line in Fig. 5a). Figure 5a presents the theoretical pressure contour lines for combinations of different sweep angles and wedge angles; the points are located on the lines, indicating that the pressure that passes through the incident wave is identical for all conditions. As the wedge angle and sweep angle increase, the static pressure that passes through the incident shocks increases.

Table 1 presents several cases of caret inlets; the inflow Mach number *M*_*a0*_ is 2.0 and the sweep angle and wedge angle are determined by the shock theory and numerical simulations. In case 1, the back-sweep angle *λ*_1_ and the effective compression angles *θ*_*1n*_ of the inner board are 30° and 9.4° and the back-sweep angle *λ*_2_ and the effective compression angles *θ*_2*n*_ of the upper board are 42° and 11.5°. The parameters of the cases *2*–5 are selected as the standard to ensure that the static pressure that passes through the two incident shocks and induced by the two boards is identical. The wave configurations for these cases can be determined by the shock polar analysis of the characteristic plane and using the method of spatial dimension reduction. Figure 5b–f shows the results of the shock polar analysis of the wave configurations for the cases 1–5 described in Table 1. The two reflected polars *R*_1_ and *R*_2_ are completely inside the incident polars *I*_1_ and *I*_2_ or are collapsed into points; this indicates that weak shock interactions will occur. Point “*s*” divides the incident polar *I* into two portions. In the portion from the bottom to point “*s*”, the flow Mach number of the characteristic plane is supersonic and in the portion from point “*s*” to the top, the flow Mach number is subsonic. When the original point of the reflected polar is in the supersonic portion, the flow passing through the incident waves is supersonic and a reflected polar is formed. When the original point of the reflected polar is in the subsonic portion, the flow passing through the incident waves is subsonic and the reflected polar collapses to a point. It should be noted that when the reflected polar is in the subsonic portion, the flow Mach number in 3D conditions may be supersonic after combining the Mach numbers of the characteristic plane and the characteristic line; this condition is very different from 2D flows. For 3D weak shock interactions, the significant characteristic is that the reflected waves are replaced by compression waves and no subsonic zones are observed, unlike in 2D conditions.

**Table 1:** Parameters of Caret inlets and theoretical results of each zone.

Case	λ_1_	θ_1n_	θ_1_	λ_2_	θ_2n_	θ_2_	P_1_	P_2_	P_m_	M_s1_	M_s2_	M_m_	θ_p1_	θ_p2_	θ_pm_
1	30	9.4	8.16	42	11.5	8.60	1.36	1.18	1.44	1.15	1.07	1.18	0.997	0.999	0.995
2	30	9.4	8.16	42	16.4	12.3	1.36	1.36	1.53	1.15	1.15	1.21	0.997	0.997	0.992
3	30	5.89	5.1	42	11.5	8.60	1.18	1.18	1.32	1.07	1.07	1.13	0.999	0.999	0.998
4	30	9.4	8.16	32	10.1	8.60	1.36	1.36	1.63	1.15	1.15	1.24	0.997	0.997	0.988
5	41	10.8	8.16	42	11.5	8.60	1.18	1.18	1.25	1.07	1.07	1.10	0.999	0.999	0.999

For the weak interactions, two incident shocks interact with each other and the surface of the Mach stem is formed. *P*_1_ and *P*_2_ are the static pressure behind the oblique waves induced by the inner board and upper board respectively; *P*_*m*_ is the static pressure behind the Mach stem. *M*_*s*1_, *M*_*s2*_, and *M*_*m*_ are the flow Mach numbers behind the two incident waves and the Mach stem. *θ*_*p*1_, *θ*_*p2*_, and *θ*_*pm*_ are the total pressure recovery coefficients behind the oblique wave of the inner board, the oblique wave of the upper board, and the Mach stem. Table 1 shows that the Mach numbers and static pressure behind the incident waves and Mach stem have little disparity, which makes the incoming flow at the entrance uniform. The performance of the total pressure recovery is excellent without considering the viscous effects and boundary layer separations and the theoretical total pressure recovery is 99% . Because the Mach number of the incoming flow is low, it decreases after projecting it onto the 2D characteristic plane in the case of a large back sweep angle. Since the weak shock interactions have excellent total pressure recovery, uniform incoming flow, and good stealth performance due to the large back sweep angle, the theory of weak shock interaction is applied to the caret inlets of the F/A-18E/F. The caret inlets have a higher total pressure recovery coefficient than common 2D inlets, as has been demonstrated in previous experiments^2^.

**Figure 6:**
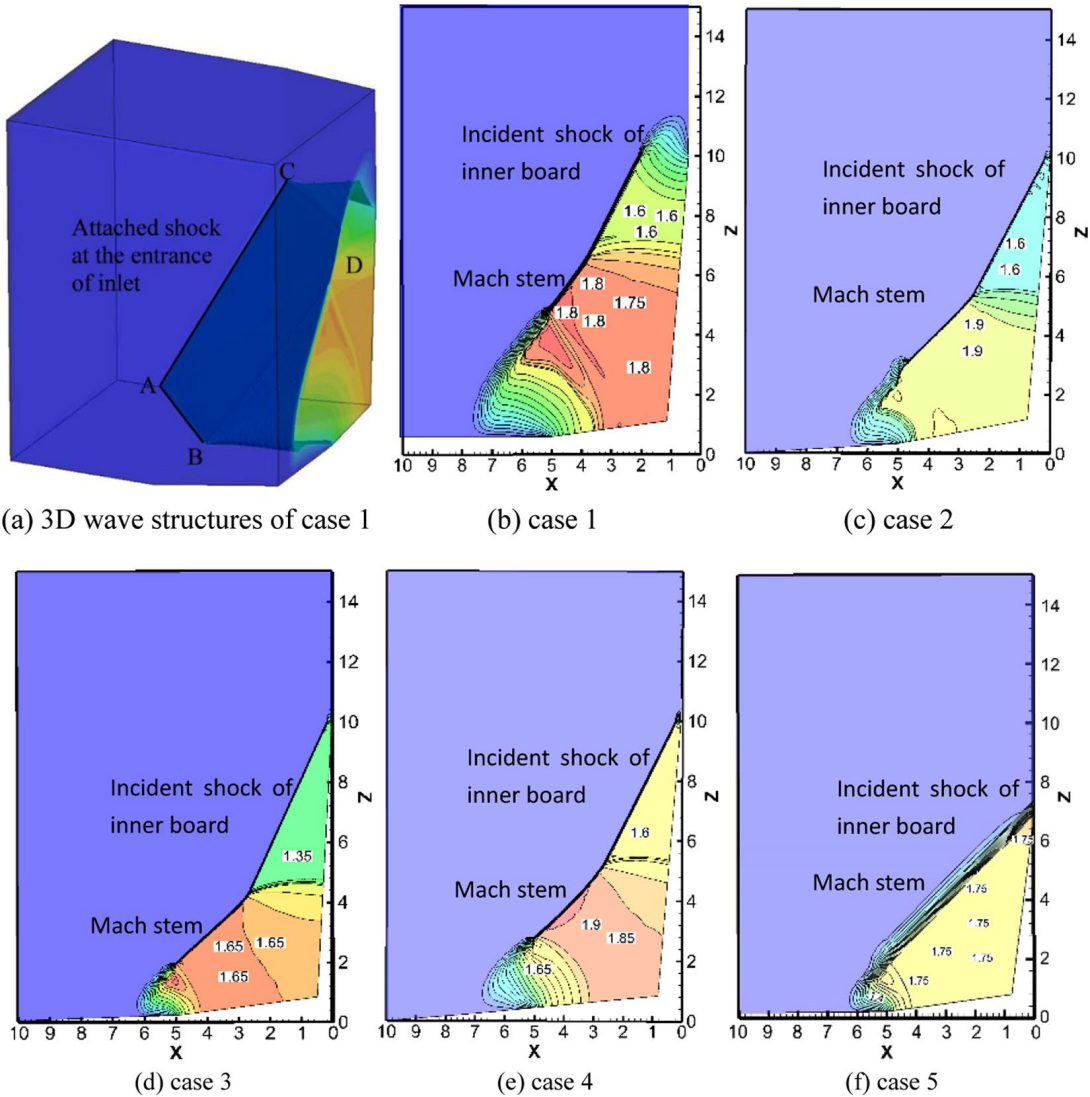
Numerical results on cross-section at *y* = 6 for cases 1–5 in Table 1.

Figure 5b shows the 3D numerical results of the caret inlet for case *1* described in Table 1. For supersonic inflow at *M*_*a0*_ = 2, two incident shocks are formed beyond the inner board and upper board of the caret inlet. The two shocks interact with each other and form a Mach stem surface between them. The Mach stem surface is almost parallel to the two incident waves, which causes the surface to looks like one attached shock. The incoming flow passes through the attached shock and provides uniform compressional flow for the inlets. Figure 5b–f shows that the wave configurations for cases *1* to 5 are weak shock interactions; the noteworthy features of weak shock interactions are the uniform flow field and high total pressure recovery coefficient. Therefore, weak shock interactions are applicable to caret inlets. Figure 6a presents the cloud charts of the pressure distribution of the caret inlet for case 1; the shock is attached at the entrance of the caret inlet *ABCD*, the contour lines are perpendicular to the direction of the incoming flow *y*. The results indicate that the incident wave induced by the inner board and the Mach stem provide compressed flow for the caret inlet and the reflected wave between the Mach stem and the incident shock of the inner board is replaced by a compression wave. The performance of the inlet is dominated by the inner board and the interacting zone induced by the inner and upper boards.

Figure 6a shows the 3D numerical results of the caret inlet for case 1 described in Table 1. For supersonic inflow at *M*_*a0*_ = 2, two incident shocks are formed beyond the inner board and upper board of the caret inlet. The two shocks interact with each other and form a Mach stem surface between them. The Mach stem surface is almost parallel to the two incident waves, which causes the surface to looks like one attached shock. The incoming flow passes through the attached shock and provides uniform compressional flow for the inlets. Figure 6(b–f) present the numerical results of the pressure distribution and the contour lines of different cross-sections for cases 1–5 described in Table 1. Since we focus on the inviscid parameters, the unit of length is dimensionless; the length ratio between the inner board in the *y*-direction and the upper board in the *x*-direction is 2:1. Because the entrance is a spatial parallelogram, the depth of the inlet in the y-direction can be calculated from the dimensions of the upper board and inner board. To investigate the effects of the weak shock interaction on the performance of the inlet, the compression part of the caret inlet is chosen as the computational zone. The numerical results indicate that the shock of the inner board and the Mach stem contribute to the compression flow to the inlet for cases 2, 3, and 5. In contrast, in case 4, there is little disparity between the sweep angles of the two boards; therefore, the shocks of the two boards and the Mach stem provide the compression flow for the inlet. When all the reflected waves of these cases are replaced by compression waves, the parameters of the flow field change little after passing through the compression waves. Therefore, the weak shock interactions provide uniform flow and excellent performance in terms of total pressure recovery.

## Conclusion

This study investigated the 3D SSI of two intersecting wedges with back-sweep angles and their applications in caret inlets. A theoretical approach called “spatial dimension reduction” was used in conjunction with numerical simulations to study the flow-field characteristics of the flows. The wave configurations and their structures, the uniformity of the flow field, and the total pressure recovery performance were discussed in detail. The results of this study provide an effective method to investigate 3D weak shock interactions, a topic that is very important to the design of caret inlets. The most notable conclusions of this study can be summarized as follows.

1. For the back-sweep angle, the theoretical and numerical investigations indicate that the wave structure is a weak shock interaction for large sweep angles and large Mach numbers; this is different with the results of previous studies. This finding is attributed to the fact that the decomposed Mach numbers of the characteristic plane are very small after taking the three-dimensional effects into account; therefore, a weak shock interaction occurs.
2. The individual Mach numbers of the characteristic plane and the combined Mach numbers of the 3D flow field are solved theoretically. As the Mach number of the incoming flow increases, the individual and combined Mach numbers behind the Mach stem and the reflected waves, the static pressure, and the total pressure loss in each zone increase monotonously. As the back-sweep angle increases, the total pressure loss decreases; this finding is very significant for the design of inlets. The combined Mach numbers in the 3D flows are the same for different back-sweep angles and the individual Mach numbers of these flows that perpendicular to the surfaces of the shock waves are different.
3. The problem of the SSI in caret inlets is a problem of weak shock interaction, which provides uniform flow and excellent performance in terms of total pressure recovery. The flows are compressed by the two incident waves of the two boards and the Mach stem formed by the interaction of the two incident waves and the reflected waves are replaced by compression waves. Since the length ratio between the inner board and upper board is 2:1, the compressed flow is provided by the incident wave of the inner board and the Mach stem.
